# Editorials

**Published:** 1891-12

**Authors:** 


### HOMŒOPATHIC PHYSICIAN,

A MONTHLY JOURNAL OF

HOMŒOPATHIC MATERIA MEDICA AND CLINICAL MEDICINE.

" If our school ever gives up the strict inductive method of Hahnemann, we are lost, and deserve only to be mentioned as a caricature in the history of medicine."—CONSTANTINE HERING.

Vol. XI. DECEMBER, 1891. No. 12.

### EDITORIALS.

A RETROSPECT.—With the present number, THE HOMŒOPATHIC PHYSICIAN completes the eleventh year of its existence. Of its record its friends may be justly proud. It was started to maintain the cause of pure Homœopathy. At the time it was founded there was no journal that could be depended upon to teach the pure doctrine and to maintain it against all foes, except that elegant quarterly, *The Organon*, published by Dr. Skinner. But Dr. Skinner suddenly ceased his admirable work and the cause of similar medicine was without an advocate. Thus there was left a field for the publication of a new journal which should set this one object of teaching Hahnemannian Homœopathy before it as its guiding principle.

The venerable Dr. Adolph Lippe, perceiving this opportunity, and feeling keenly the absence of a publication that should lead the profession into the paths indicated by Hahnemann, determined to start a new journal that should be the worthy successor of *The Organon*.

Looking about among his younger friends for a suitable editor, he selected Dr. Edmund J. Lee. Thus was founded THE HOMŒOPATHIC PHYSICIAN, a journal with *a mission* and a journal that contained within itself the elements of success because it had *a mission*.

For nine years Dr. Lee conducted the journal with consummate ability and the hearty approval of Dr. Lippe, until his health gave out and he relinquished it to the management of the present editor and his associate, Dr. George H. Clark. The latter, after a service of nearly two years, has withdrawn, leaving it entirely in the hands of the writer of this article. Our readers will miss the brilliant and incisive editorials of Dr. Clark that have enlivened its pages and extended its influence, but we expect to enrich our pages from time to time with articles from the same gifted pen.

In saying that this journal is successful, it must not be understood that it yields large pecuniary profits, for it has not succeeded in such a result. But it has achieved an influence for itself second to none in the journalism of our school, simply because it has remained true to its mission. Even its bitterest enemies know well that if they wish to learn what is the current of thought among pure homœopathists they must consult its pages.

It will continue in the future as in the past to maintain its medical principles, and the record it has made in the past will be a guarantee for the future.

It now remains for the profession to show their appreciation of it, to give it their indorsement by promptly paying their annual dues and by contributing articles to its pages. Every practitioner of Homœopathy should realize that the maintenance of this journal is the maintenance of his own standing in the community. As pure Homœopathy faithfully applied will achieve more cures than mixed or rational methods of practice, so those who avail themselves of it must constantly increase the number of their patients. That they may be enabled to properly apply the method, they must continually read a journal that keeps it vividly before their minds. Such a journal is THE HOMŒOPATHIC PHYSICIAN that should number every man in the profession among its friends and supporters, and have not a single enemy, for none can have an honest grievance against it.

W. M. J.

Is It Homœopathy or Isopathy?—In the November number at page 425, Dr. Swan in his article upon "Homœopathy or Isopathy," says: "They cannot realize the change that potentization makes in the drug, changing it from an isopathic substance to a homœopathic remedy."

With all due regard to Dr. Swan, who is numbered among the friends of the editor, it seems proper to take issue with him upon this point. It is impossible to understand how simply potentizing any substance can make it homœopathic to any disease condition whatever.

Any drug in the materia medica may be homœopathic to any conceivable disease condition when the totality of the symptoms of the one agrees with the totality of the symptoms of the other. On the other hand, no drug in the materia medica can be homœopathic to any disease condition unless its symptoms do so agree.

Under what circumstances does a drug become homœopathic to a sick condition? The answer obviously is that when it has been proved and the provings have been recorded, we have the information just *what* is its sphere of action; just *what* it will do. Then when we compare this record with the symptoms of the patient we perceive that it is or it is not in agreement with them. Accordingly we say that the drug is or it is not homœopathic to them; and that is only another way of saying that it is or it is not *similar*. How then can a morbose product be homœopathic to a sick condition simply by the process of potentization? How do we know that it is homœopathic when we have no record of provings of it with which we may compare the symptoms of the patient?

It is known to the writer, of course, that Dr. Swan claims that the symptoms of a disease are a virtual proving of the morbose product of that disease. He has maintained this dogma with great courage and perseverance in the face of a storm of opposition and even reviling. But it may be submitted that many diseases have more than one morbose product, and the morbose product varies from time to time. Which one of these products will he select; at what stage of its development, and on what grounds? In addition to this difficulty, it would seem that such a procedure compels us to recognize *disease as an entity* with invariable manifestations instead of a sick condition with varying character. This relegates us to the domain of the old school of medicine, whom we hold to be in error.

There can be no objection to Dr. Swan's introducing morbose products into our materia medica if he will but *prove* them. That is the corner-stone of all medical advancement, and its importance is virtually acknowledged by the regular school. Without it we float on a sea of doubts having neither rudder nor compass to guide us. W. M. J.

